# Broadband Graphene-PbS Heterostructure Photodetector with High Responsivity

**DOI:** 10.3390/nano15030207

**Published:** 2025-01-28

**Authors:** Xinbo Mu, Jinbao Su, Wenjuan Zhou, Pengying Chang, Jun Deng, Ying Liu, Zhengtai Ma, Yiyang Xie

**Affiliations:** Key Laboratory of Optoelectronics Technology, Beijing University of Technology, Ministry of Education, Beijing 100124, China; muxinbo5268@163.com (X.M.); b202272009@bjut.edu.cn (W.Z.); pychang@bjut.edu.cn (P.C.); dengsu@bjut.edu.cn (J.D.); dkliuying@bjut.edu.cn (Y.L.); mazhengtai@bjut.edu.cn (Z.M.); xieyiyang@bjut.edu.cn (Y.X.)

**Keywords:** graphene, PbS, photodetector, responsivity

## Abstract

Graphene-based photodetectors exhibit relatively low spectral absorption and rapid recombination of photogenerated carriers, which can limit their response performance. On the other hand, nanostructured lead sulfide (PbS) demonstrates a wide spectral absorption range from visible to near-infrared light. High-quality and evenly distributed PbS nanofilms were synthesized by chemical bath deposition and were applied to a graphene-PbS heterostructure photodetector. The heterostructure creates an inherent electric field that extends the lifetime of photogenerated carriers, leading to enhanced device response. We achieved a high-responsivity graphene-PbS photodetector by combining the high carrier mobility of graphene and the strong infrared absorption of PbS. The photodetector exhibits a responsivity of 72 A/W at 792 nm and 5.8 A/W at 1550 nm, with a response time of less than 20 ms. The optimized device features a broad spectral response ranging from 265 nm to 2200 nm.

## 1. Introduction

Graphene is a two-dimensional crystal composed of a single layer of hexagonally arranged carbon atoms. Since 2004, it has attracted widespread attention from researchers due to its high carrier mobility, exceptional mechanical properties, good thermal conductivity, and high transparency [1,2,3]. Based on these properties, graphene has extensive potential applications in sensing, imaging, spintronics, and flexible wearable devices [4,5,6,7]. Since graphene is a zero-bandgap material, incident light across a wide range of wavelengths—from ultraviolet to terahertz—should be able to generate a significant photocurrent in graphene. This enables the potential use of graphene in multiple spectral bands for various applications. Although graphene exhibits an extremely broad absorption spectrum, its absorption efficiency varies significantly across different wavelengths [8]. Most reported graphene-based photodetectors exhibit sensitivity only to specific wavelength ranges, thereby limiting the applicability of these devices to a certain extent. In addition to the narrow detectable wavelength range, single-layer graphene shows a low light absorption efficiency of only 2.3% in some cases, which may not meet the essential requirements for a photodetector [9]. Although doping can greatly enhance the absorption efficiency for certain wavelength bands, it can also reduce the carrier mobility of graphene, adversely affecting the response speed of graphene photodetectors. Additionally, graphene demonstrates a rapid photoresponse, with electron–hole pair generation occurring in less than 50 fs upon light incidence [10]. However, due to the short lifetime of photo-generated carriers in graphene, the responsivity of graphene photodetectors is only in the order of mA/W [11].

Currently, researchers use heterostructures in the majority of graphene detectors to effectively separate photo-generated carriers through the built-in electric field, thereby extending the lifetime of photo-generated carriers [12]. Yang et al. integrated graphene with high-mobility InGaAs to form a graphene-InGaAs heterojunction, achieving a responsivity of 7.66 A/W at a 1550 nm wavelength [13]. Jeong et al. reported a fivefold enhancement in photocurrent at a 980 nm wavelength by combining graphene with highly absorbent PbS quantum dots compared to PbS quantum dots alone [14]. However, the fabrication process of PbS quantum dots is relatively complex and their response wavelength is relatively narrow. Emmanuel K et al. proposed a chemical bath deposition method to prepare PbS thin films on graphene surfaces, forming graphene-PbS heterojunctions and demonstrating transient photocurrent characteristics at a 532 nm wavelength [15]. Nevertheless, the direct deposition of PbS films on graphene surfaces via chemical bath deposition unavoidably brings about doping that impacts the mobility of graphene [16].

In this paper, we present a broadband graphene-PbS photodetector. The unique feature of this device’s structure is that the graphene is in contact with the PbS only in the middle region of the channel. This ensures that no current flows through the PbS, but rather, an internal electric field is formed between the PbS and the graphene to capture photogenerated holes. Consequently, the lifetime of the photogenerated carriers is extended, achieving higher responsivity. Additionally, PbS thin films were synthesized by the chemical bath deposition method, which ensures material compatibility with various substrates, enhancing device applicability. By controlling the ratio of chemical reagents, bath temperature, and growth time, we obtained uniform PbS thin films with significant light absorption in the near-infrared region. The graphene-PbS photodetector showed a responsivity of 5.8 A/W under 1550 nm laser illumination. Furthermore, it demonstrated a detectable response across the spectral range from 265 nm to 2200 nm. Compared to the graphene-Si photodetectors mentioned in the literature [17], the responsivity is significantly improved.

## 2. Materials and Methods

### 2.1. Material and Device Fabrication

The fabrication process of the device involves several key steps. First, a PbS film with an approximate thickness of 500 nm is deposited onto a glass substrate using the chemical bath method, with a rotation speed of 180 r/min and a constant temperature of 30 °C. Subsequently, the PbS film undergoes annealing in a N_2_/O_2_ (50 sccm/50 sccm) atmosphere at 400 °C for 20 min using a rapid annealing furnace. Following this, a 300 nm SiO_2_ layer, used as the dielectric layer, is deposited on the surface of the PbS film using PECVD. Photolithography and etching processes are then employed to expose the underlying PbS, and metal electrodes are deposited on the SiO_2_ surface. Single-layer graphene is transferred onto the device’s surface using a wet etching transfer method and then patterned using photolithography and etched by oxygen plasma. The wet etching transfer of graphene is carried out in the following steps. Initially, a layer of polymethyl methacrylate (PMMA) is used to coat the graphene surface. The copper foil substrate of the graphene is etched using a solution of copper sulfate and hydrochloric acid (CuSO_4_:HCl:H_2_O = 5 g:20 mL:120 mL). Once the copper foil is corroded, the graphene is transferred to the deionized water and soaked repeatedly. Finally, the transferred graphene is treated with acetone and isopropanol to remove the PMMA. The channel area of the device measures 2 × 10^−5^ cm^2^.

### 2.2. Device Characterization

The quality of the graphene was assessed using Raman spectroscopy (HORIB LabRAM HR—Paris, FR, Franceat) a wavelength of 532 nm and a beam diameter of 2 μm. The spectra were recorded using a spectrophotometer (Hitachi U-4100—Tokyo, JP, Japan). Electrical and optoelectronic tests were conducted at room temperature using a semiconductor characterization system (Agilent B1500A—Santa Clara, CA, USA) with a probe station. The probe station was enclosed in a light-tight box to minimize ambient light interference. The laser power was measured using a power meter (PM400, Thorlabs Company—Newton, NJ, USA). The response of the photodetector was evaluated using lasers of different wavelengths as light sources.

## 3. Results and Discussion

**Figure 1:**
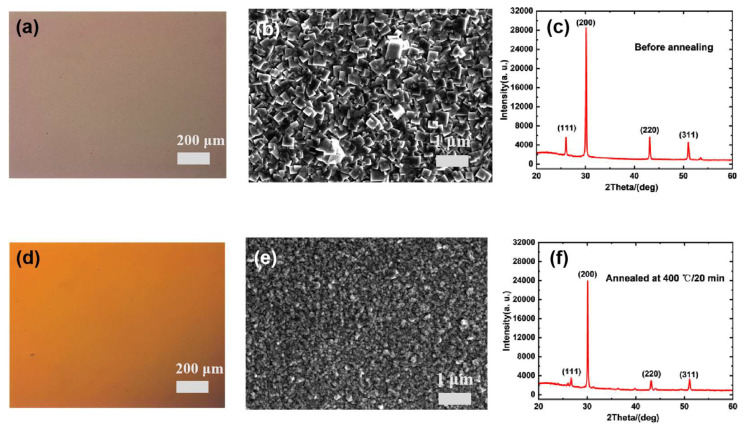
(**a**) Optical micrograph of PbS before annealing. (**b**) SEM image of PbS before annealing. (**c**) XRD pattern of PbS before annealing. (**d**) Optical micrograph of annealed PbS. (**e**) SEM image of annealed PbS. (**f**) XRD pattern of annealed PbS.

High-quality thin films play a crucial role in the performance of devices. Therefore, we conducted characterization and analysis of PbS thin films. Figure 1a,d show the optical images of the PbS film before and after annealing at 400 °C. It can be observed that the color of the PbS surface turned from coffee to orange, which indicates that the photosensitivity of PbS is improved after annealing [18]. Figure 1b,e display the scanning electron microscope images of the PbS film before and after annealing. After annealing, it can be seen that the film’s surface morphology transformed from cubic grains to uniformly distributed pebble-like grains, and the average grain size decreased from 500 nm to approximately 250 nm due to film oxidation [19]. Additionally, cracks appeared on the annealed film surface, which resulted from the mismatch in thermal expansion coefficients between the PbS film and the glass substrate during the rapid cooling process after annealing [20]. Figure 1c,f show the XRD patterns of the PbS films before and after annealing. Both samples indicate that the PbS (200) crystal plane is their preferred orientation. When comparing the relative diffraction peak intensities of different crystal directions, the peak intensity ratios I_220_/I_200_ and I_311_/I_200_ before annealing are 0.198 and 0.160, and those after annealing are 0.123 and 0.137, respectively. The results indicate that the (200) orientation of the film is enhanced after annealing. After annealing, some new diffraction peaks are attributed to the formation of the PbO·PbSO_4_ phase due to the presence of oxygen.

**Figure 2:**
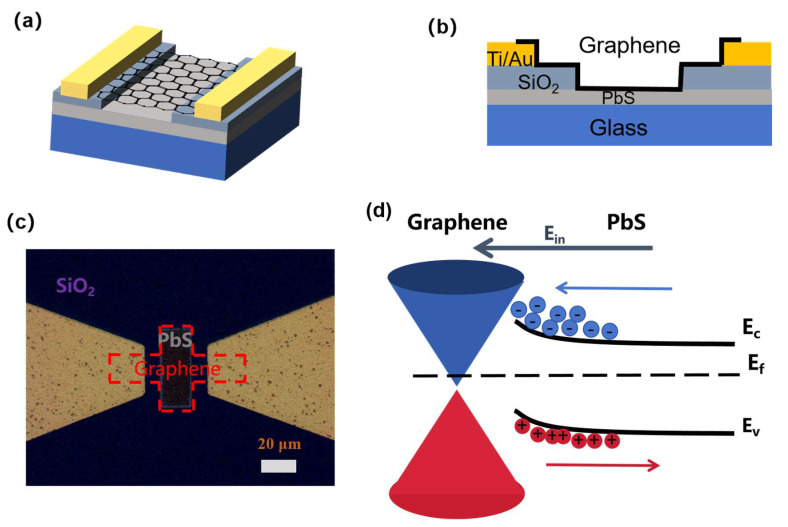
(**a**) The top view and (**b**) a cross-section image of the graphene-PbS photodetector. (**c**) An optical image of the device’s surface. (**d**) A band diagram of the graphene-PbS heterostructure. E_C_, E_V_, and E_F_ represent the conduction band minimum, valence band maximum, and Fermi level of PbS, respectively. Blue arrows indicate the direction of the built-in electric field E_in_.

Figure 2a,c illustrate the top view and a microscopic image of the graphene-PbS photodetector, respectively. Figure 2b depicts a schematic diagram of the graphene-PbS photodetector. A graphene-PbS heterojunction is constructed in the central region of the graphene. The reflectance spectrum of PbS, shown in Supplementary Figure S1, indicates that the bandgap of PbS is approximately 0.95 eV. The energy band diagram of the graphene-PbS heterojunction is displayed in Figure 2d. The interaction between graphene and PbS generates a built-in electric field, which promotes the effective separation of electron–hole pairs and significantly reduces the recombination rate of photogenerated carriers, consequently extending their lifetime. The net photocurrent in a photoconductive detector can be written as follows: $I_{ph}=gALe\cdot G$, where g is the generation rate of excess carriers, L is the channel length, A is the cross-sectional area, e is the elementary charge, and G is the gain. The gain factor G is defined as the ratio of the carrier lifetime $\tau_{n}$ to the transit time $\tau_{t}$, such that $G=\tau_{n}/\tau_{t}$ [21]. Thus, extending the lifetime of photogenerated carriers may enhance the photoconductive gain of the device, thereby improving its responsivity.

**Figure 3:**
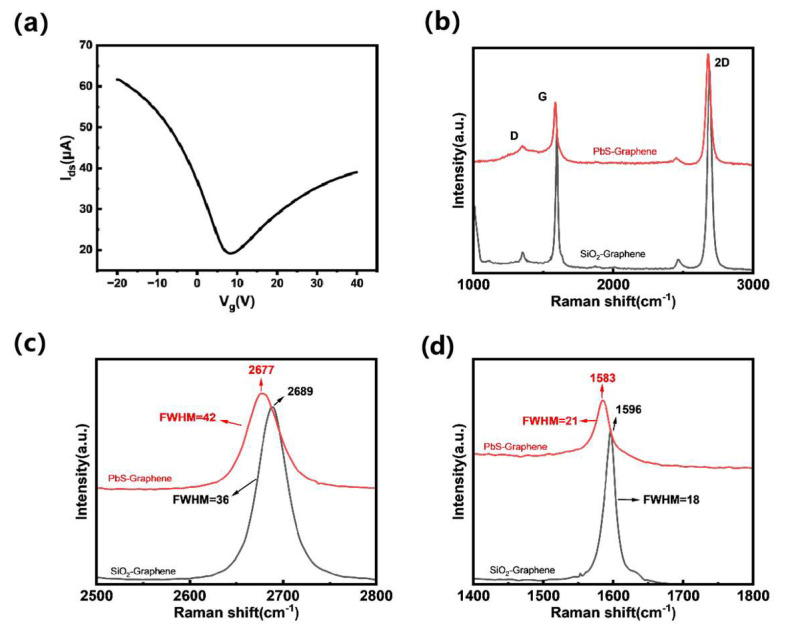
(**a**) The transfer characteristics of the graphene field-effect transistor. (**b**) The Raman spectra of graphene (red curve: Raman spectrum of graphene on PbS surface, black curve: Raman spectrum of graphene on SiO_2_ surface). (**c**) The shift in the 2D peak position and the changes in its FWHM. (**d**) The shift in the G peak position and the changes in the FWHM of graphene.

We fabricated a graphene field-effect transistor (FET) (Supplementary Figure S2) and measured its electrical properties. As shown in Figure 3a, the Dirac point of graphene is approximately located at +7 V. This indicates that the graphene undergoes p-type doping due to the adsorption of oxygen and water molecules when exposed to air. However, placing the graphene under vacuum conditions can restore its pristine state [22,23,24,25,26]. Based on the FET mobility formula $\mu =(L/WC_{ox}V_{d})(∆I_{d}/∆V_{g})$, where $C_{ox}$ is the gate capacitance per unit area of the gate dielectric, the carrier mobility of graphene was estimated to be approximately 800 cm^2^V^−1^s^−1^.

As illustrated in Figure 3c,d, upon transferring graphene onto the PbS surface, the full width at half maximum (FWHM) of both the G and 2D peaks in the Raman spectra increased significantly. Specifically, the FWHM of the G peak increased from 18 cm^−1^ to 21 cm^−1^, accompanied by a redshift of 13 cm^−1^. Meanwhile, the FWHM of the 2D peak rose from 36 cm^−1^ to 42 cm^−1^, with a redshift of 12 cm^−1^. These changes suggest that, in addition to strain effects, the transfer of graphene onto the PbS surface led to an increase in the electron concentration due to the significant difference in work functions between the two materials. However, considering the initial p-type doping background of graphene exposed to air and the subsequent changes in the optoelectronic response of the graphene-PbS device, the overall doping nature of graphene continues to be of the p-type [27,28,29].

**Figure 4:**
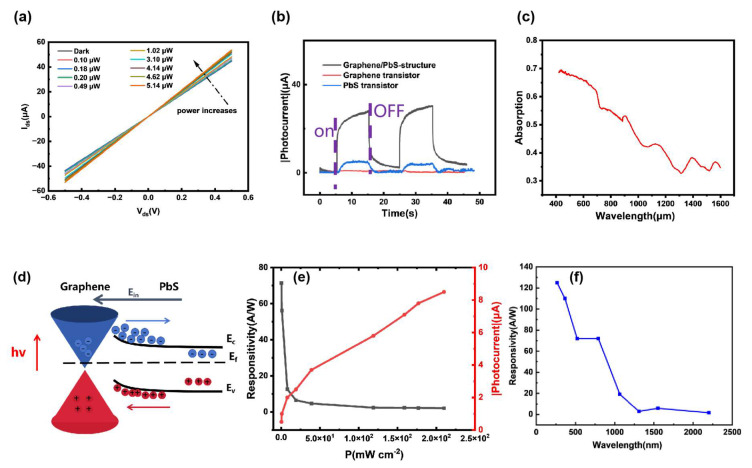
(**a**) The output characteristics of devices under 792 nm laser irradiation at different laser power levels. (**b**) The dynamic output characteristics under 1064 nm (675 mWcm^−2^) laser irradiation: traditional graphene photodetector (red), PbS photodetector (blue), and graphene-PbS photodetector (black) with a field-effect structure. (**c**) The absorption spectra of graphene-PbS. (**d**) The band structure of the graphene-PbS heterojunction under illumination. (**e**) The device responsivity and photocurrent as a function of incident optical power. We investigated the dependence of photocurrent on device power under 792 nm laser (V_DS_ = 0.5 V) irradiation. (**f**) The device responsivity across different wavelengths (265, 365, 520, 792, 1064, 1310, 1550, and 2200 nm).

The graphene-PbS photodetector was measured using a laser source spanning from ultraviolet to near-infrared wavelengths. As shown in Figure 4a, a significant photoconductive change was observed under 792 nm laser illumination. As shown in Figure 4b, under illumination with a 1064 nm laser of the same wavelength, the device exhibited faster and more pronounced response signals during the on-off cycles compared to the graphene photodetector (Supplementary Figure S2) and PbS photodetector (Supplementary Figure S4). Notably, under 1064 nm laser illumination, the response rate of the graphene-PbS device increased by three orders of magnitude compared to the graphene device. Figure 4c shows the absorption spectra of graphene-PbS at different wavelengths. The dynamic photoresponse of the device was measured at 265, 365, 520, 1310, 1550, and 2200 nm laser sources (Supplementary Figure S3). As shown in Figure 4d, when light is applied to the graphene-PbS surface, photo-generated carriers are generated in both graphene and PbS. Under the influence of the built-in electric field, the electron–hole pairs are separated, with electrons migrating towards PbS and holes towards graphene. This leads to an increase in the hole concentration and electrical conductivity of p-type graphene, resulting in a stronger response from the device. Figure 4e demonstrates that as the optical power gradually increases, the photocurrent approaches saturation, resulting in a gradual decrease in responsivity. As shown in Figure 4f, the device demonstrates responsivities exceeding 1 A/W across a wavelength range of 265 nm to 2200 nm. The responsivities for laser wavelengths of 265, 365, 520, 1064, 1310, and 2200 nm are 125, 110, 71.9, 19.2, 2.9, and 1.6 A/W, respectively. It is challenging for photodetectors made by graphene to achieve this level of performance. Its capacity for broad-spectrum response extends the potential uses for this device across various applications.

The measurement results indicate that the graphene-PbS photodetector demonstrates significant responsivity, exceeding 19 A/W under a bias of 0.5 V and illumination by a 1064 nm laser. Furthermore, under laser irradiation at wavelengths of 265, 365, 520, and 792 nm, the responsivity remains above 70 A/W, with a response speed of less than 10 ms. The photoconductive gain of the device is given by the formula G=R×(hc/λe) [30], where G represents the photoconductive gain, h is Planck’s constant, c is the speed of light, e is the electron charge, and R is the responsivity. Under the aforementioned conditions, the gain is calculated to be 109.7. This formula can be rewritten as $G=\tau_{n}/\tau_{t}$. Assuming the same transit time between source and drain electrodes as in traditional graphene field-effect transistors (Supplementary Figure S2), the lifetime of photogenerated carriers in graphene in our device is extended by approximately 900 times. The performance of the graphene-PbS photodetector was assessed in comparison to other graphene-based photodetectors in the near-infrared region (Table 1). The graphene-PbS photodetector demonstrates higher responsivity at 1550 nm. This is due to PbS absorbing incident photons to generate electron–hole pairs. The built-in electric field drives the holes generated in PbS into graphene, leading to changes in the electrical conductivity of graphene and a significant increase in its responsiveness.

## 4. Conclusions

This study introduces a graphene-PbS heterostructure photodetector that efficiently captures holes, thanks to the built-in electric field formed between graphene and PbS. This configuration extends the lifetime of photocarriers and enhances device responsivity. The detector exhibits a responsivity level greater than 1 A/W across the spectral range from 265 nm to 2200 nm. Specifically, under illumination from lasers at 792 nm and 1550 nm, the device demonstrates high responsivity levels of 72 and 5.8 A/W, with optical gains of 109.7 and 7.03, respectively, and a response time of less than 20 ms. We believe that this graphene-PbS photodetector holds promise for advancing high-performance photodetectors in the future.

## Figures and Tables

**Table 1:** A comparison of the optical responsivity of our device with that of previous graphene-based 1550 nm photodetectors.

Device Structure	Responsivity	Wavelength	Reference
Graphene-PbS	5.8 A/W	1550 nm	This work
Graphene-PbS QDs	183 mA/W	1550 nm	[31]
Graphene-PbS QDs	0.16 A/W	1550 nm	[32]
Graphene-PbS QDs	0.15 A/W	1550 nm	[33]
Graphene-Si	39.5 mA/W	1550 nm	[34]
Graphene-Si	0.43 A/W	1550 nm	[35]
Graphene p-n junction	1.4 A/W	1550 nm	[36]
Graphene-AlO-InGaAs	1.32 A/W	1550 nm	[37]
Graphene-Ge	2.02 A/W	1550 nm	[38]

## Data Availability

The data presented in this study are available on request from the corresponding author.

## Supplementary Materials

The following supporting information can be downloaded at: https://www.mdpi.com/article/10.3390/nano15030207/s1, Figure S1: Tauc plot of PbS annealed at 400 °C (inset: reflectance spectra of annealed PbS); Figure S2: Structure schematic and optical image of graphene field effect transistor; Figure S3: Dynamic photoresponse of device under laser sources of varying wavelengths; Figure S4: Structure schematic and optical image of PbS field effect transistor.
