# Supplementary material for: Broadband Graphene-PbS Heterostructure Photodetector with High Responsivity
Source: Nanomaterials (Basel). 2025 Jan 28;15(3):207. doi: 10.3390/nano15030207 (PMC11819821; doi:10.3390/nano15030207)
Supplement: Supplementary file 1 [file nanomaterials-15-00207-s001.zip › nanomaterials-3440089-supplementary.pdf]

Supplementary Materials for

Broadband graphene-PbS heterostructure photodetector with high responsivity

Xinbo Mu [1], Jinbao Su [1], Wenjuan Zhou [1], Pengying Chang [1], Jun Deng [1], Ying Liu [1], Zhengtai Ma [1] and Yiyang Xie [1,*]

[1]Key Laboratory of Optoelectronics Technology, Beijing University of Technology, Ministry of Education, Beijing 100124, China
*Correspondence: xieyiyang@bjut.edu.cn(Y.X.)

1. The reflectance spectrum of PbS

We measured the reflectance spectrum of PbS prepared by chemical bath deposition and calculated the bandgap of PbS using the Tauc plot method. The Tauc plot of PbS thin films annealed at 400 °C is shown in Fig. S1, with insets depicting the reflectance spectra of PbS at different wavelengths.

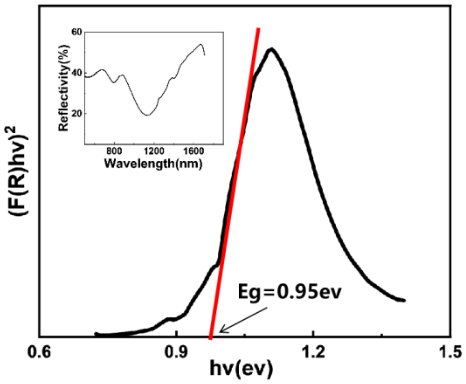

Supplementary Figure S1.The Tauc plot of PbS annealed at 400°C (inset: Reflectance spectra of annealed PbS).

2. Structure schematic and optical image of Graphene field effect transistor

To calculate the mobility of graphene and the responsivity of graphene detectors, we fabricated graphene field-effect transistors as depicted in Fig. S2.

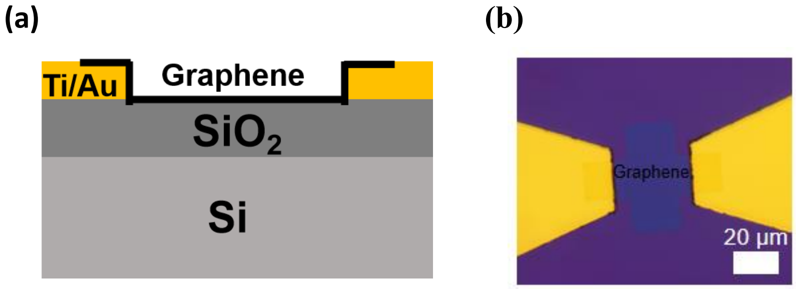

Supplementary Figure S2 Structure schematic and optical image of Graphene field effect transistor.

3. The dynamic optical response of graphene-PbS photodetectors under laser sources of different wavelengths.

We measured the dynamic photoresponse of the graphene-PbS photodetectors under laser sources at wavelengths of 265, 365, 520, 1310, 1550 and 2200 nm, respectively.

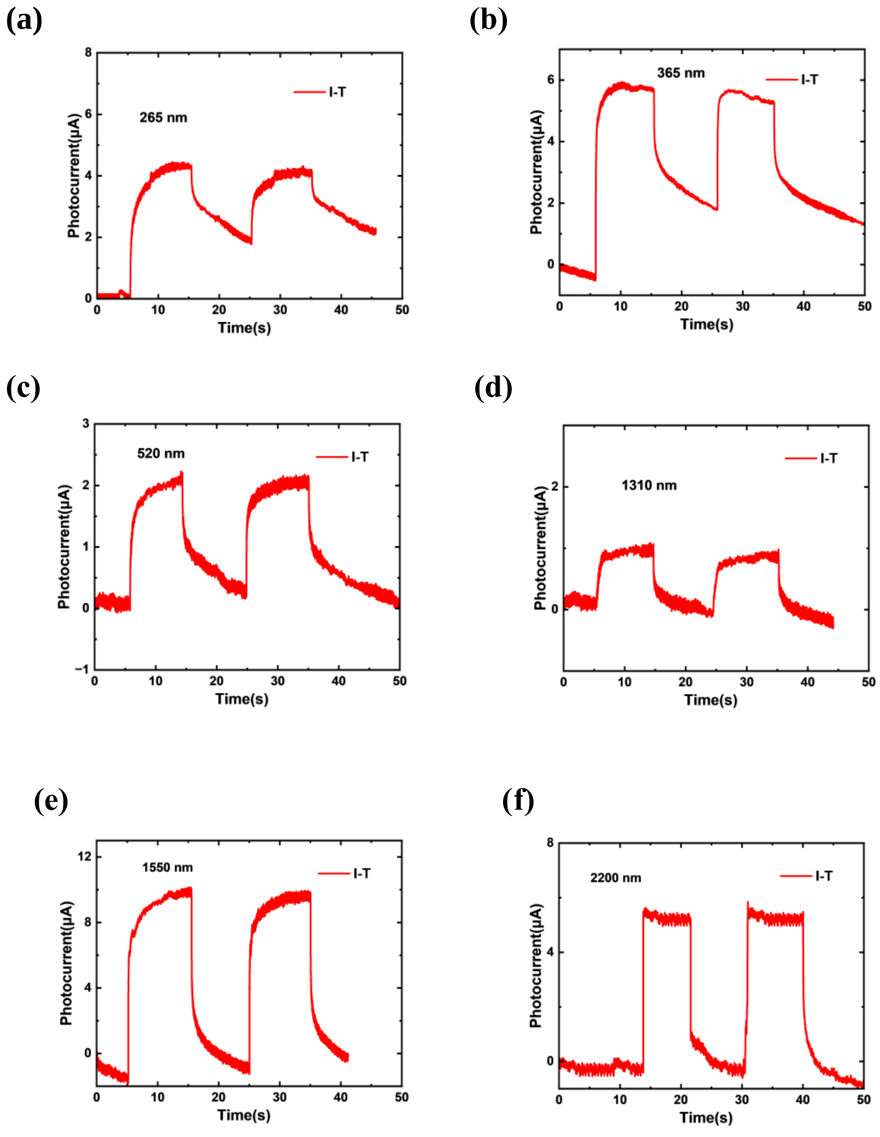

Supplementary Figure S3 Time-dependent photocurrent measurement (a) 265 nm (2 mWcm-2); (b) 365 nm (2.6 mWcm-2); (c) 520 nm (1.39 mWcm-2); (d) 1310 nm (1.68 mWcm-2); (e) 1550 nm (85.7 mWcm-2); (f) 2200 nm (160 mWcm-2) laser irradiation.

4. **Structure schematic and optical image of PbS field effect transistor**

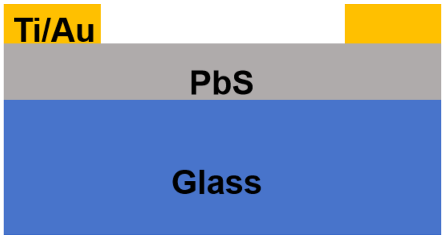

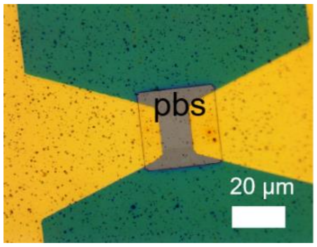

Supplementary Figure S4 Structure schematic and optical image of PbS field effect transistor.
